# Multimodal Shared Autonomy for Heavy-Load UAV Operations with Physics-Aware Cooperative Control

**DOI:** 10.3390/s26061997

**Published:** 2026-03-23

**Authors:** Xu Gao, Jingfeng Wu, Yuchen Wang, Can Cao, Lihui Wang, Bowen Wang, Yimeng Zhang

**Affiliations:** 1 Construction Branch, State Grid Shaanxi Electric Power Co., Ltd., Xi’an 710005, China; 2 Shaanxi Power Transmission and Transformation Engineering Company Limited, Xi’an 710003, China; 3 School of Microelectronics, Xidian University, Xi’an 710126, China

**Keywords:** multimodal interaction, shared autonomy, heavy-load UAV, human–machine cooperation, physics-aware control

## Abstract

Heavy-load unmanned aerial vehicles (UAVs) are increasingly being applied in logistics, infrastructure installation, and emergency response missions, where complex payload dynamics and unstructured environments pose significant challenges to safe and efficient operation. Conventional manual teleoperation interfaces, such as dual-joystick control, impose a high cognitive workload and provide limited support for expressing high-level operator intent, while fully autonomous solutions remain difficult to deploy reliably under real-world uncertainty. To address these limitations, this paper proposes the Multimodal Fusion Cooperation Network (MFCN), an end-to-end shared autonomy framework that integrates speech commands, visual gestures, and haptic cues through cross-modal feature fusion to infer operator intent in real time. The fused intent representation is translated into dynamically feasible control commands by a cooperative control policy with embedded physics-aware constraints to suppress payload oscillations and ensure flight stability. Extensive semi-physical simulations and real-world experiments demonstrate that the MFCN significantly improves the task success rate, positioning accuracy, and payload stability while reducing the task completion time and operator cognitive workload compared with manual, unimodal, and heuristic multimodal baselines.

## 1. Introduction

Heavy-payload unmanned aerial vehicles (UAVs) are rapidly evolving from conventional sensing platforms into aerial work systems capable of transporting and positioning loads that range from tens to hundreds of kilograms [1,2,3,4,5]. This capability enables time-critical or hard-to-access operations such as material delivery and emergency response [6], lifting bulky items [7], medical logistics [8], and the placement or installation of infrastructure components in cluttered outdoor scenes [4,9]. In these missions, the UAV is not only required to track a trajectory but to do so while managing the coupled dynamics of a suspended or articulated payload and maintaining safety margins in the presence of wind, occlusions, and perception uncertainty.

Despite significant progress in autonomy, heavy-load missions remain difficult to execute reliably under a purely manual or purely autonomous paradigm. From a control perspective, the UAV must regulate its six-degree-of-freedom motion while suppressing payload oscillations that can be modeled as pendulum-like dynamics [10], becoming particularly challenging under disturbances and modeling uncertainty [11,12]. From a perception and human operation perspective, these tasks are often performed outdoors, where lighting changes, viewpoint shifts, and partial occlusions degrade sensing and complicate decision-making [13]. Consequently, practical systems frequently rely on a human operator to provide task guidance, while autonomy is expected to stabilize the system and enforce safety constraints in real time.

However, the dominant interface for heavy-load UAV teleoperation remains dual-joystick control, where the operator issues low-level commands such as velocity or attitude rate references [14,15,16]. While such interfaces offer fine-grained authority, they also impose a substantial cognitive workload because the operator must simultaneously regulate vehicle motion, compensate for payload swing, and maintain situational awareness under time pressure [17,18]. This burden was also reflected in our real flight study: joystick teleoperation yielded NASA-TLX scores of 72.4±5.1 under 2–3 m/s wind and 81.7±6.3 under 4–6 m/s wind, whereas the MFCN reduced these values to 49.3±3.9 and 55.8±4.5, respectively. Moreover, joystick inputs provide a low-dimensional channel that poorly conveys higher-level intent such as “grasp that container” or “align with platform B” [19]. Situational awareness is further limited because operators typically rely on narrow first-person video streams without haptic/force cues, and onboard sensing can degrade under adverse conditions [20,21]. These limitations motivate interaction paradigms that increase the expressiveness and robustness of intent communication while reducing operator workloads.

Shared autonomy provides a principled framework for human–machine cooperation in which a human specifies goals and resolves ambiguities and autonomy assists with execution, stabilization, and safety [22,23]. However, applying shared autonomy to heavy-load UAVs introduces unique complications: payload coupling tightens safety constraints, the cost of misinterpretation is high, and intent must be inferred under real-world uncertainty. A key bottleneck is the interaction modality—the system needs a reliable channel to interpret intent in real time, while being resilient to missing or corrupted sensory inputs.

Early human–UAV interaction approaches often used a single modality (e.g., gestures [24] or speech [25]) and have shown that natural interfaces can reduce the barrier to control. Nonetheless, unimodal signals are vulnerable to ambiguity and environmental interference [26,27]: speech may be semantically underspecified without spatial grounding, while gestures can be occluded or confused in complex scenes. These challenges motivate multimodal interaction, which leverages complementary information across channels to disambiguate intent and improve robustness [28,29]. In robotics, combining voice, gestures, and haptic cues has been repeatedly shown to improve reliability and naturalness in human–robot collaboration [30,31].

In this paper, we introduce the Multimodal Fusion Cooperation Network (MFCN), an end-to-end framework for the cooperative operation of heavy-load UAVs. The MFCN synchronizes and encodes speech, gesture, and haptic signals; fuses them through cross-modal temporal reasoning to infer a latent intent representation; and maps this intent—together with the UAV–payload state—to dynamically feasible control commands under physics-aware safety constraints. The proposed approach aims to (i) increase the expressiveness of operator intent communication, (ii) reduce the operator workload by shifting effort from low-level stabilization to high-level instruction, and (iii) improve robustness when one modality becomes unreliable (e.g., gesture occlusion or acoustic noise). We validate the MFCN via semi-physical simulations and real-world experiments and additionally benchmark the perception modules on public datasets to assess generalization.

The main contributions are as follows:

- We formulate heavy-load UAV shared autonomy as a multimodal intent-conditioned control problem and propose the MFCN to infer operator intent from synchronized speech, gesture, and haptic streams through learned cross-modal temporal reasoning rather than rule-based modality arbitration.
- We introduce a physics-aware cooperative control policy that converts the inferred intent and the UAV–payload state into dynamically feasible actions while explicitly regularizing payload swing energy growth and unsafe behavior.
- We establish a multi-stage evaluation protocol spanning semi-physical simulation, HIL validation, public benchmark perception tests, and real flight experiments, showing consistent improvements in task success, positioning accuracy, swing suppression, and operator workload over manual, unimodal, heuristic multimodal, and autonomy-only baselines.

## 2. Related Work

### 2.1. Shared Autonomy for Human–UAV Cooperation

Shared autonomy integrates human decision-making with autonomous execution to improve performance and safety in interactive robotic systems [22,32]. In UAV operation, this typically means that humans provide goals or coarse guidance while autonomy performs stabilization, motion generation, or constraint enforcement [33]. A persistent challenge is intent communication: mapping human instructions to machine-executable actions with minimal ambiguity and minimal operator effort [34]. Conventional interfaces such as dual joysticks or graphical user interfaces are precise but demanding when tasks require concurrent navigation and disturbance rejection [14,17]. Recent work explores intent-aware assistance mechanisms [19,23], but many approaches rely on a single modality (e.g., voice or pose), which can be insufficient under noise, occlusion, or context changes [27]. In contrast, we treat intent as a multimodal construct and design a learned fusion mechanism that explicitly reasons over complementary cues from speech, gestures, and haptics.

### 2.2. Heavy-Load UAV Control with Suspended Payloads

Heavy-load UAVs carrying suspended payloads exhibit coupled and underactuated dynamics; payload swing can be amplified by aggressive maneuvers and environmental disturbances [10,35]. A rich body of work addresses oscillation suppression and robust trajectory tracking using geometric control and feedback linearization [36], nonlinear MPC [37], and disturbance observer-based methods [38], often assuming fully autonomous execution with predefined references. Manual teleoperation, in contrast, can generate dynamically inconsistent commands that induce oscillations or violate safety margins, especially when the operator must react under uncertainty [39,40]. Our approach complements control-theoretic advances by embedding swing-aware safety objectives into a learning-based cooperative policy that translates human intent into stable, feasible actions, building on recent progress in DRL for swing suppression [41].

Beyond geometric and MPC-based approaches, robust nonlinear control strategies based on backstepping and sliding-mode design have also been widely studied for UAVs operating under uncertainty [42,43]. These methods often combine observer-based disturbance estimation with Lyapunov-guided control synthesis to guarantee bounded tracking errors in the presence of model mismatch and external perturbations [43,44]. Representative examples include backstepping sliding-mode control with disturbance compensation for quadrotors carrying suspended loads [44] and backstepping sliding-mode control using a super-twisting observer for trajectory tracking under partial state measurements [45]. Such controllers offer formal stability guarantees and strong disturbance rejection, but they typically assume that a feasible reference trajectory or command source is already available. In contrast, our work addresses a complementary layer of the problem: inferring high-level operator intent from heterogeneous human inputs and translating it into dynamically feasible shared autonomy actions while still incorporating payload dynamics-aware safety regularization during policy learning.

### 2.3. Multimodal Interaction and Fusion in Robotics

Multimodal interfaces that combine speech, gestures, and haptics can improve robustness and naturalness in human–robot interaction [28,46,47]. Speech offers symbolic commands and semantic constraints [48], gestures convey spatial and directional grounding [29,49], and haptics enable bidirectional cues related to contact, urgency, or corrective feedback [20,31,50]. Many systems use heuristic fusion (e.g., gating speech with gesture confirmation, rule-based modality switching) [51], which may fail to capture nonlinear dependencies across modalities and evolving task contexts. Moreover, much of the literature targets ground robots [52] or small UAVs [46] and does not explicitly incorporate the real-time safety constraints and payload-coupled dynamics of heavy-load aerial operations. The MFCN addresses these gaps by learning an end-to-end mapping from multimodal human input to dynamically feasible control outputs, using cross-modal attention to model correlations and physics-aware constraints to ensure safety.

Related questions of interaction asymmetry and latent human intent have also been studied in autonomous driving, especially for heavy vehicles and socially coupled decision-making. Recent studies on socially game-theoretic lane changes for autonomous heavy vehicles [53], the elimination of uncertainty in driver social preferences [54], and social predictive intelligent driver models [55] show that safe interaction benefits from explicitly modeling uncertainty and asymmetric behavior, rather than relying on fixed rules. Although developed for ground vehicles, these studies support our design choice to treat UAV shared autonomy intent inference as a context-dependent and uncertainty-aware fusion problem rather than a static modality-switching problem.

## 3. Methodology

**Figure 1:**
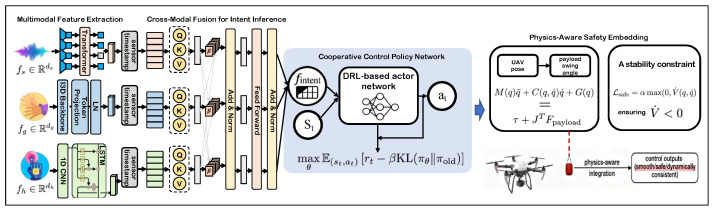
Overall architecture of the proposed Multimodal Fusion Cooperation Network (MFCN).

This section introduces the proposed Multimodal Fusion Cooperation Network (MFCN), an end-to-end framework designed for safe and efficient human–machine cooperation in heavy-load UAV operations. As illustrated in Figure 1, the MFCN consists of three tightly coupled components: (i) a multimodal perception module that encodes speech, gesture, and haptic inputs into synchronized latent features; (ii) a cross-modal fusion module that infers operator intent through temporal and semantic reasoning across modalities; and (iii) a cooperative control policy that maps the inferred intent and UAV–payload state to dynamically feasible control commands under physics-aware safety constraints.

### 3.1. Framework Overview

At each time step *t*, the system receives multimodal operator inputs together with the current UAV–payload state. The multimodal observation is defined as

(1) $$X_{t}=\left\{x_{t}^{\left(s\right)},x_{t}^{\left(g\right)},x_{t}^{\left(h\right)}\right\},$$

where $x_{t}^{\left(s\right)}$, $x_{t}^{\left(g\right)}$, and $x_{t}^{\left(h\right)}$ denote speech, gesture, and haptic signals, respectively. These inputs are processed by modality-specific encoders and subsequently fused to produce a latent intent representation $f_{\mathrm{intent}}$. The cooperative control policy then generates a control command

(2) $$a_{t}=\pi_{\theta }\left(f_{\mathrm{intent}},s_{t}\right),$$

where $s_{t}$ denotes the UAV–payload state. The policy is optimized to accomplish the operator-specified task while maintaining flight stability and suppressing payload oscillations.

### 3.2. Multimodal Perception and Feature Encoding

Each input modality is encoded into a compact feature representation using a dedicated encoder architecture, allowing the system to capture complementary semantic, spatial, and physical cues.

Speech Encoding. Raw speech signals are processed using a pretrained speech representation backbone (e.g., wav2vec 2.0 or HuBERT), followed by a lightweight Transformer encoder. This module extracts high-level semantic features $f_{s}\in R^{d_{s}}$ that capture command intent such as lift, align, or descend.

Gesture Encoding. Visual gesture inputs, represented as RGB or depth image sequences, are processed using a spatiotemporal convolutional network to capture motion dynamics. A temporal Transformer further refines these features, yielding gesture embeddings $f_{g}\in R^{d_{g}}$ that encode spatial direction and target-related cues.

Haptic Encoding. Haptic signals are acquired from an in-house two-axis force-feedback side-stick (FFJ-2D prototype). The raw stream is sampled at 200 Hz and consists of joystick deflection and force-feedback channels $\left[\delta_{x},\delta_{y},F_{x},F_{y}\right]$. Before encoding, the deflection channels are normalized by the full-scale joystick range, the force channels are clipped at ±20 N and scaled to [−1,1], and all channels are standardized using training set statistics. The normalized sequence is then downsampled to 50 Hz and processed by a 1D CNN-LSTM encoder to produce haptic features $f_{h}\in R^{d_{h}}$ that capture the corrective intent, interaction intensity, and urgency cues during operation.

All modality-specific embeddings are temporally synchronized using sensor timestamps and projected into a shared latent space prior to fusion.

### 3.3. Cross-Modal Fusion for Intent Inference

To infer operator intent from heterogeneous inputs, the MFCN employs a cross-modal attention fusion block with two stacked Transformer layers. Each modality embedding is first projected into a shared latent space of dimension $d_{\mathrm{model}}=256$ as

(3) $$z_{m}=\mathrm{LN}\;\left(W_{m}f_{m}+b_{m}+e_{m}\right),$$

where $e_{m}$ denotes a modality/time embedding and LN(·) is layer normalization. The synchronized token set $Z=[z_{s};z_{g};z_{h}]$ is then processed by two Transformer blocks with 4 attention heads, feedforward width $d_{\mathrm{ff}}=512$, and dropout 0.1. The intent embedding is obtained by mean-pooling the final hidden states, i.e.,

(4) $$f_{\mathrm{intent}}=\mathrm{Pool}\;\left({\mathrm{Transformer}}_{\mathrm{cross}}\left(Z\right)\right).$$

This design allows the network to model cross-modal dependencies without hard modality-switching and yields a fixed-length intent representation for the downstream policy. When modalities conflict, the cross-attention module redistributes weights softly according to temporal consistency, rather than forcing a deterministic switch to a single channel.

The fused intent representation encodes both semantic objectives (e.g., “grasp the object”) and execution-related constraints (e.g., motion direction or interaction intensity). During training, the fusion module is supervised using aligned intent labels or task trajectories, minimizing

(5) $$L_{\mathrm{fusion}}={∥f_{\mathrm{intent}}-f_{\mathrm{target}}∥}_{2}^{2},$$

where $f_{\mathrm{target}}$ is obtained from expert demonstrations or synchronized task annotations.

### 3.4. Cooperative Control Policy

The cooperative control policy $\pi_{\theta }$ maps the inferred intent $f_{\mathrm{intent}}$ and the UAV–payload state $s_{t}$ to a control command $a_{t}$. We implement $\pi_{\theta }$ as a deep reinforcement learning actor network trained with a hybrid reward function:

(6) $$r_{t}=r_{\mathrm{task}}-\lambda_{1}{∥{\dot{\theta }}_{p}∥}^{2}-\lambda_{2}E_{\mathrm{ctrl}}-\lambda_{3}d_{\mathrm{collision}},$$

where $r_{\mathrm{task}}$ measures the task completion quality, ${\dot{\theta }}_{p}$ denotes the payload swing rate, $E_{\mathrm{ctrl}}$ represents the control effort, and $d_{\mathrm{collision}}$ penalizes unsafe proximity to obstacles. All reward terms are normalized to comparable ranges using training set statistics. The final weights are $\lambda_{1}=0.40$, $\lambda_{2}=0.05$, and $\lambda_{3}=0.80$.

Policy optimization is performed using proximal policy optimization (PPO), solving

(7) $$\max_{\theta }E_{\left(s_{t},a_{t}\right)}\left[r_{t}-\beta \mathrm{KL}\left(\pi_{\theta }∥\pi_{\mathrm{old}}\right)\right].$$

We selected PPO because it yields more stable optimization and lower performance variance than SAC under the physics-aware regularization adopted in this work. All experimental results reported in this paper therefore reflect the use of PPO only.

This design allows the policy to translate high-level human intent into dynamically feasible and stable UAV maneuvers.

### 3.5. Physics-Aware Safety Regularization

To ensure real-world deployability, physics-aware safety constraints are embedded into the learning process. The coupled UAV–payload dynamics are modeled as

(8) $$M\left(q\right)\ddot{q}+C\left(q,\dot{q}\right)\dot{q}+G\left(q\right)=\tau +J^{⊤}F_{\mathrm{payload}},$$

where $q={\left[p,\eta ,\theta_{x},\theta_{y}\right]}^{⊤}$ contains the UAV position, attitude, and payload swing angles. The airframe mass and inertia parameters were obtained from the platform CAD model and bench measurements, the cable length was measured directly as l=1.5m, and the residual swing-damping coefficients were identified from free-swing decay experiments using least-squares fitting.

A Lyapunov-inspired regularization term is incorporated during training,

(9) $$L_{\mathrm{safe}}=\alpha \max\;\left(0,\dot{V}\left(q,\dot{q}\right)\right),$$

where $V(q,\dot{q})$ is defined as the mass-normalized payload swing energy,

(10) $$V\left(q,\dot{q}\right)=\frac{1}{2}l^{2}\left({\dot{\theta }}_{x}^{2}+{\dot{\theta }}_{y}^{2}\right)+gl\left(2-\cos\theta_{x}-\cos\theta_{y}\right).$$

Using the mass-normalized form makes the safety term independent of the exact payload mass. Together with domain randomization over payloads of 5, 10, 20, and 40kg during training, this enables the policy to adapt to mass variations through the observed swing state and vehicle response without requiring the true mass as an explicit input.

### 3.6. Training and Implementation Details

The MFCN is trained using a combination of semi-physical simulation and real-world datasets. The simulation environment models stochastic disturbances, including wind and sensor noise, as well as domain randomization over payload masses of 5–40kg. The perception, fusion, and control modules are pre-trained independently and subsequently fine-tuned jointly using imitation learning and reinforcement learning objectives. All networks are optimized using Adam with a learning rate of $1\times 10^{-4}$. On the onboard Jetson AGX Orin (NVIDIA Corporation, Santa Clara, CA, USA) the complete inference loop runs at 30Hz with mean end-to-end latency of 24.7ms and 95th-percentile latency of 29.8ms. The average per-cycle latency is 7.8ms for speech feature extraction, 10.6ms for gesture encoding, 1.1ms for haptic encoding, 1.4ms for timestamp synchronization and buffering, 1.9ms for cross-modal fusion, and 1.9ms for policy inference and safety regularization.

## 4. Experiments

This section evaluates the proposed Multimodal Fusion Cooperation Network (MFCN) using semi-physical simulation, hardware-in-the-loop (HIL) validation, and real-world flight experiments. We assess task success, efficiency, payload stability, robustness to perception degradation, and the operator cognitive workload, and we compare the MFCN with manual, autonomous, and shared autonomy baselines.

### 4.1. Experimental Setup

UAV Platform and Payload Configuration. We employ a heavy-load hexarotor platform with a maximum payload capacity of 40kg, equipped with an onboard Jetson Orin (NVIDIA Corporation, Santa Clara, CA, USA) [56] for real-time inference in the MFCN pipeline. A standardized suspended payload of 20kg is attached via a 1.5m cable, introducing pronounced coupled UAV–payload swing dynamics. The operator interface consists of the FFJ-2D force-feedback side-stick for haptic input, a depth camera for gesture acquisition [57], and a noise-canceling microphone for speech commands.

Semi-Physical Simulation and HIL. A Gazebo/ROS-based semi-physical simulator [58] models full 6-DOF UAV–payload dynamics. In HIL mode, a Pixhawk flight controller [59] is integrated to verify timing, signal flow, and the 30Hz real-time execution constraint of the policy. To reflect realistic operating conditions, we introduce stochastic disturbances, including payload mass variations (∈{5,10,20,40}kg), wind gusts up to 8m/s, and temporary perception degradation (e.g., partial occlusions). Each scenario is repeated for 1000 episodes (20k episodes in total).

Real-World Flight Tasks. We evaluate three representative heavy-load operational tasks: (i) Align-and-Place (hover-to-target placement), (ii) Navigate-and-Deliver (long-distance transit with suspended payload), and (iii) Dock-to-Platform (final-stage precision landing and alignment). The results reported in Table 1 are aggregated averages across these tasks. All experiments were conducted under relevant safety regulations.

Participants and Procedure. Twelve operators participated in the real flight study: 4 novice users (<20 h of multirotor flight experience), 4 intermediate users (20–100 h), and 4 experienced users (>100 h). Before data collection, all participants received a standardized 60 min training session comprising a 15 min safety/system briefing, 15 min interface familiarization, and 30 min supervised practice with the MFCN interface and the baseline joystick workflow. Each participant completed all three tasks under JT, AO, and MFCN in both wind bands, and NASA-TLX was recorded after each method × wind block.

Public Benchmark Evaluation. To assess the generalization of perception components beyond the UAV domain, we isolate the gesture, speech, and haptic encoders from the closed-loop control pipeline and evaluate them on public benchmark datasets. Only minor adaptations (input preprocessing and output heads) are applied to match the dataset requirements, while the core encoder architectures are kept unchanged, enabling a controlled and standardized evaluation.

### 4.2. Baselines and Control Architectures

To ensure a fair comparison, all learning-based shared autonomy methods employ the same actor–critic DRL backbone [60]. This design isolates the effects of (i) the intent representation and (ii) the multimodal fusion strategy, as the policy capacity and training procedures are matched across methods.

JT (Manual Teleoperation) is a fully human-controlled baseline, where low-level velocity commands are issued via a joystick without intent inference or autonomy assistance.

AO (Autonomous Optimization) is a fully autonomous baseline implemented as nonlinear MPC with explicit swing suppression objectives and without human input. We selected MPC as the sole autonomy-only baseline because it is a strong and widely used model-based controller for suspended-load UAV systems and provides a transparent reference for comparing shared autonomy against fully autonomous execution. The controller uses a horizon N=20, sampling interval Δt=0.1s, state-tracking weight $w_{p}=15$, velocity weight $w_{v}=2$, swing penalty $w_{s}=10$, control-effort weight $w_{u}=0.1$, and terminal weight 30. These parameters were selected through validation across wind, occlusion, and payload mass variations while keeping the solver within the 30Hz real-time budget.

UM (Unimodal Shared Autonomy) conditions the policy on a single encoded modality (e.g., gesture-only), without learned cross-modal interaction.

HM (Heuristic Multimodal) uses rule-based fusion. Speech confidence is defined as the top-1 posterior probability of the speech classifier, gesture confidence is the temporally averaged top-1 posterior over a 15-frame window, and haptic confidence is the sigmoid output of an urgency/override head computed from the haptic encoder features. The switching thresholds are $c_{s}\geq 0.70$, $c_{g}\geq 0.65$, and $c_{h}\geq 0.60$. When the confidence gap between the two highest-scoring modalities is smaller than Δc=0.15, the previous fused command is held for one cycle to avoid chattering. Otherwise, the highest-confidence modality dominates, with speech defining the symbolic command, gesture providing spatial direction, and haptic input modulating the corrective intensity.

MFCN (Ours) integrates multimodal observations using a cross-modal Transformer fusion module [61] and trains a DRL policy with physics-aware constraints [62] for swing-aware, safe control.

### 4.3. Quantitative Results in Simulation

Table 2 shows that the MFCN achieves the best overall performance across all metrics, including the highest success rate (92.4%) and the lowest positional error (0.18m). Relative to manual teleoperation (JT), the MFCN reduces the task completion time by 28.3% and decreases the maximum payload swing by 57.8%. Compared with the strongest baseline (HM), the MFCN further improves the efficiency and accuracy, yielding an additional 18.5% reduction in the task completion time and a 33.3% improvement in positional accuracy. These results indicate that learned cross-modal fusion provides more reliable intent inference than rule-based strategies, leading to more stable and efficient control.

**Figure 2:**
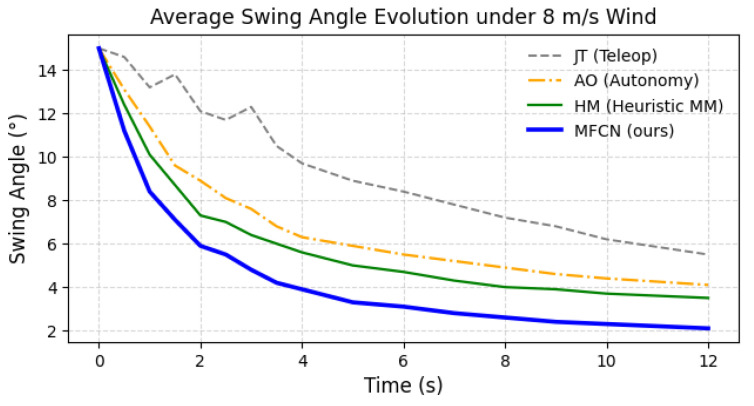
Average payload swing evolution under moderate wind (8m/s). MFCN exhibits reduced oscillation amplitude and faster damping compared to baselines.

Figure 2 further illustrates the stability improvements, where the MFCN achieves the fastest damping and the lowest oscillation magnitude. For Table 2, statistical significance was assessed using one-way repeated-measures ANOVA over the 20 scenario-level aggregates, followed by Holm-corrected paired *t*-tests for post hoc comparisons. The main effect of the method was significant for the success rate, task completion time, positional error, maximum swing, and swing decay (all adjusted p<0.001). In particular, the MFCN significantly outperformed HM and AO in the key closed-loop metrics reported in Table 2.

### 4.4. Module-Specific Evaluation on Public Datasets

To evaluate whether the MFCN’s perceptual components generalize beyond the UAV operational domain, we benchmark the gesture and speech encoders on representative public datasets under standardized settings.

Gesture Encoder. We evaluate the gesture encoder on AUTH UAV Gesture [63] and UAV-Gesture [24]. AUTH UAV Gesture contains 4930 videos across six gesture categories and is challenging due to viewpoint variation and high inter-class similarity among several gestures. UAV-Gesture contains 119 UAV-captured videos with 13 command gestures performed by 10 subjects, providing complementary diversity with fewer samples. For a controlled comparison, we resample the input sequences to match each dataset’s spatial resolution, fix the temporal window to T=15 frames, and replace the classifier head to match the number of classes. As shown in Table 3, our encoder achieves 82.6% and 96.3% accuracy on AUTH UAV Gesture and UAV-Gesture, respectively, outperforming the representative baselines.

Speech Encoder. We benchmark the speech encoder on Google Speech Commands v2 [67] and LibriSpeech [68]. Speech Commands provides short command phrases suitable for evaluating instruction recognition, while LibriSpeech tests robustness across speakers and recording conditions. For each dataset, we match the sampling rates, adjust the output head for keyword classification (Speech Commands) or CTC-style decoding support (LibriSpeech), and retrain under the same optimization settings. As shown in Table 4, our encoder achieves F1 scores of 0.97 on Speech Commands and 0.91 on LibriSpeech, outperforming the strong baselines. These results suggest that the learned acoustic representations are transferable and robust to domain variation.

### 4.5. Real-World Results and Cognitive Load

**Figure 3:**
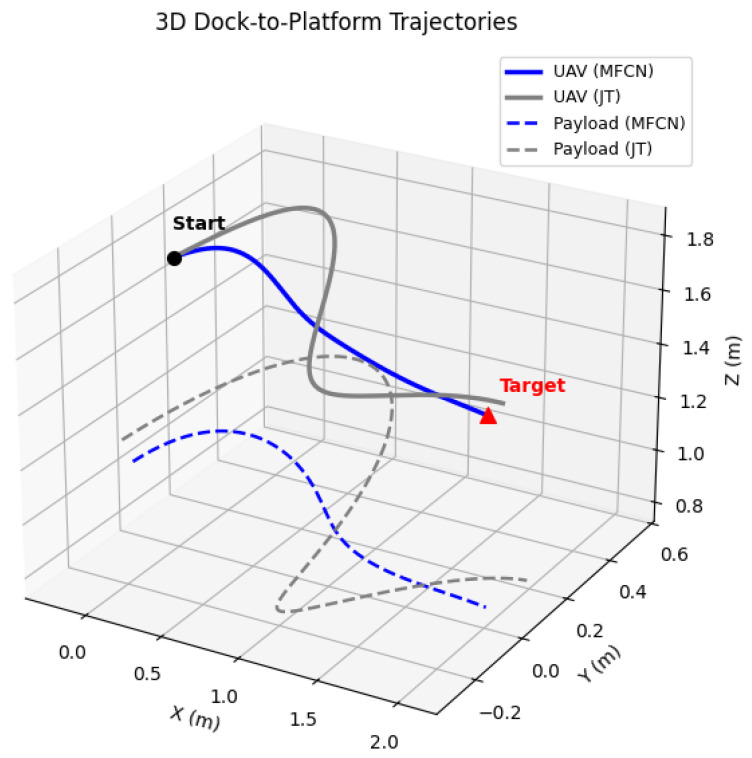
Three-dimensional trajectories of the UAV (solid) and suspended payload (dashed) in the Dock-to-Platform task. MFCN achieves smoother approach and reduced overshoot compared with JT.

Real-world flights corroborate the simulation results. During the Dock-to-Platform task, Figure 3 shows that the MFCN produces smoother trajectories and faster damping of payload swing than JT. Under moderate wind (4–6m/s), the MFCN maintains an 83.1% success rate, whereas that of AO drops to 67.1%. We therefore revise the interpretation to emphasize two factors, rather than attributing the robustness to intent guidance alone: firstly, human intent remains available when onboard perception or the scene geometry makes autonomy-only target selection ambiguous; secondly, the physics-aware policy converts high-level guidance into swing-suppressed commands rather than raw manual corrections. NASA-TLX was analyzed using a two-way repeated-measures ANOVA with factors method and wind, followed by Holm-corrected paired *t*-tests. The workload reduction of the MFCN relative to JT was significant under both 2–3m/s and 4–6m/s winds (both adjusted p<0.001), and the MFCN’s result also remained significantly lower than that of AO under 4–6m/s wind (adjusted p<0.001).

### 4.6. Ablation Study and Feature Importance

Table 5 highlights the contribution of each component. Removing physics-aware regularization increases the maximum swing and reduces the success rate, indicating that the safety term is important in shaping the policy toward swing-suppressed behaviors. Removing any single modality degrades the intent accuracy and downstream task performance, with the largest drops observed when removing haptic or gesture inputs, suggesting that haptics provide interaction/urgency cues while gestures provide spatial grounding required for precision.

**Figure 4:**
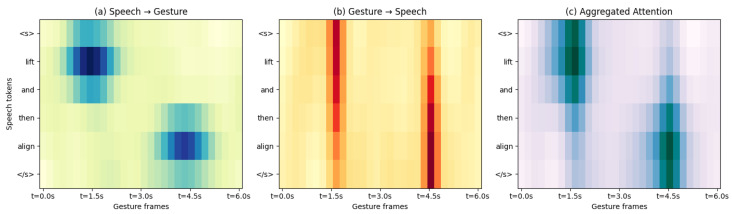
Cross-modal attention visualization during the “lift and align” task. The attention maps illustrate temporal grounding between speech tokens and gesture frames, reflecting context-dependent intent interpretation.

Figure 4 visualizes the bidirectional attention patterns between speech tokens and gesture frames during a “lift and align” task. The model initially emphasizes tokens related to “lift” and progressively shifts attention toward “align” as stabilization is achieved, while gesture-to-speech attention exhibits a slight temporal lag consistent with human response dynamics. The aggregated attention patterns indicate that the MFCN captures context-aware cross-modal dependencies rather than relying on fixed modality rules.

### 4.7. Robustness Under Perception Degradation

**Figure 5:**
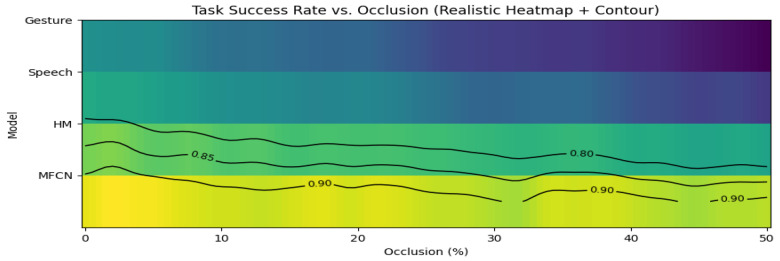
Task success rate under increasing visual occlusion severity for different control strategies. MFCN shows improved robustness due to cross-modal redundancy.

Figure 5 reports the task success rates under increasing visual occlusion severity. The performance of the MFCN degrades gracefully as occlusion increases and it consistently outperforms the HM and unimodal baselines, indicating that cross-modal redundancy mitigates failure when gesture observations are partially corrupted. For example, at 30% occlusion, the MFCN’s result decreases from 92.4% to 80.3%, while that of the gesture-only policy drops to 55.7%.

## 5. Discussion

The results from the simulation, HIL, and real-world experiments collectively demonstrate that the MFCN improves both performance and usability for heavy-load UAV operation.

### 5.1. Impact of Multimodal Fusion on Shared Autonomy

The MFCN achieves higher intent accuracy and better closed-loop stability than unimodal and heuristic multimodal baselines. This supports the premise that multimodal inputs provide complementary information: speech conveys symbolic task constraints, gestures provide spatial grounding, and haptics add interaction intensity and corrective cues. Cross-modal attention enables the system to exploit these cues adaptively as the task context changes.

In conflicting input situations, the learned fusion module does not rely on a fixed priority rule. Instead, speech primarily anchors symbolic task semantics, gesture refines spatial grounding, and haptic cues modulate urgency or the corrective intensity; cross-modal attention then reweights these streams according to temporal consistency. This soft arbitration helps to avoid abrupt policy changes when operators produce transiently contradictory commands under stress.

### 5.2. Role of Physics-Aware Regularization

Ablation studies show that removing physics-aware regularization increases oscillation and reduces the success rate, confirming that embedding payload dynamics into learning objectives is essential in suppressing swing and maintaining stability. This is particularly important for heavy-load platforms, where energy coupling between the vehicle and the payload can amplify oscillations under aggressive or inconsistent commands.

### 5.3. Operator Workload and Trust

Real-world results indicate that the MFCN reduces the NASA-TLX scores substantially, suggesting that cooperative autonomy shifts the operator from continuous low-level stabilization to higher-level intent expression. Informal operator feedback further suggests improved predictability and responsiveness when multimodal cues are available, which is important for trust in shared autonomy systems.

### 5.4. Robustness and Generalization

The MFCN maintains high task success under partial perception degradation, demonstrating robustness through modality redundancy. Benchmark evaluations on public datasets also indicate that the learned perceptual components transfer beyond the UAV-specific domain, supporting broader applicability.

### 5.5. Limitations and Future Work

The current implementation assumes calibrated sensors and reliable time synchronization, which may not always hold in field deployment. In addition, speech understanding is based on predefined command vocabularies, and scaling to open-vocabulary interaction remains an open challenge. Finally, our current focus is a single human–UAV pair; extending to multi-agent cooperative aerial manipulation will require explicit intent disambiguation across multiple humans and robots. These directions motivate future work on adaptive synchronization, open-vocabulary multimodal intent grounding, and multi-agent coordination.

## 6. Conclusions

This work proposes the MFCN as a practical shared autonomy framework for heavy-load aerial operations in which multimodal human intent and physics-aware control are tightly integrated. Beyond the specific tasks studied here, the results suggest a broader design principle for aerial robotics: operators should communicate high-level goals through expressive multimodal channels, while autonomy should handle low-level stabilization, swing suppression, and safety enforcement. This division of responsibility is especially important for heavy-load platforms operating in uncertain outdoor environments, where neither low-level teleoperation nor autonomy-only control is sufficiently robust on its own. The proposed framework therefore offers a concrete step toward deployable shared autonomy for aerial construction, logistics, and emergency response missions.

## Figures and Tables

**Table 1:** Real flight evaluation (aggregated over three tasks) under two wind conditions. Values are mean ± SD over 12 operators. Lower TTC and NASA-TLX are better. NASA-TLX was collected after each method × wind block. The best results are highlighted in bold.

Method	Wind (m/s)	SR (%)	PosErr (m)	MaxSwing (°)	TTC (s)	NASA-TLX
JT	2–3	82.0 ± 3.5	0.36 ± 0.07	12.1 ± 1.8	33.7 ± 4.1	72.4 ± 5.1
JT	4–6	40.5 ± 7.9	0.54 ± 0.12	17.8 ± 2.5	41.3 ± 5.8	81.7 ± 6.3
AO	2–3	88.4 ± 3.1	0.29 ± 0.05	8.4 ± 1.3	30.9 ± 3.7	68.2 ± 4.9
AO	4–6	67.1 ± 5.2	0.38 ± 0.08	11.2 ± 1.9	35.8 ± 4.5	70.1 ± 5.5
**MFCN**	2–3	**95.2** ± **2.1**	**0.17** ± **0.03**	**5.8** ± **0.9**	**25.6** ± **3.2**	**49.3** ± **3.9**
**MFCN**	4–6	**83.1** ± **3.4**	**0.21** ± **0.04**	**7.1** ± **1.1**	**29.8** ± **3.8**	**55.8** ± **4.5**

**Table 2:** Comparison of overall performance in simulation (averaged over all scenarios). Bold indicates best results.

Method	SR (%)	TTC (s)	PosErr (m)	MaxSwing (°)	SwingDecay (s)	CtrlEffort
JT (Teleop)	56.2 ± 4.1	38.5 ± 5.2	0.42 ± 0.08	14.7 ± 2.1	9.1 ± 1.4	1.00 ± 0.12
AO (Autonomy)	78.1 ± 2.5	34.7 ± 3.9	0.29 ± 0.05	8.9 ± 1.3	6.5 ± 0.9	0.88 ± 0.07
UM (Gesture-Only)	74.3 ± 3.1	36.2 ± 4.1	0.31 ± 0.06	9.8 ± 1.5	7.0 ± 1.1	0.91 ± 0.09
HM (Heuristic MM)	81.5 ± 2.8	33.9 ± 3.5	0.27 ± 0.04	8.2 ± 1.2	6.0 ± 0.8	0.87 ± 0.06
**MFCN (Ours)**	**92.4** ± **1.9**	**27.6** ± **2.8**	**0.18** ± **0.03**	**6.2** ± **0.8**	**4.3** ± **0.6**	**0.79** ± **0.05**

**Table 3:** Gesture recognition performance on AUTH UAV Gesture and UAV-Gesture datasets. The best results are highlighted in bold.

Method	AUTH UAV Gesture (%)	UAV-Gesture (%)
P-CNN [64]	-	91.9
DD-Net [65]	74.2	91.5
MLP [66]	76.2	94.8
**Ours**	**82.6**	**96.3**

**Table 4:** Comparison of F1 scores on two public speech benchmarks. The best results are highlighted in bold.

Dataset	Ours	BC-ResNet [69]	KWS [70]
Speech Commands v2 [67]	**0.97**	0.95	0.96
LibriSpeech [68]	**0.91**	0.86	0.89

**Table 5:** Ablation study in simulation. For each variant, we removed one component from the full MFCN. IntentAcc denotes the top-1 intent classification accuracy of the fused intent head on synchronized 1 s multimodal windows from held-out validation episodes, where a prediction is counted as correct when the inferred intent label matches the annotated task command. The best results are highlighted in bold.

Variant	SR (%)	MaxSwing (°)	PosErr (m)	IntentAcc (%)	Comment
Full MFCN	**92.4**	**6.2**	**0.18**	**94.1**	—
w/o Physics loss	83.7	9.1	0.25	93.5	Policy becomes less stable
w/o Haptic	85.3	7.4	0.23	83.0	Reduced urgency/interaction cue
w/o Speech	88.0	6.9	0.20	88.5	Weaker symbolic instruction channel
w/o Gesture	86.7	7.3	0.22	86.1	Reduced spatial grounding
Early Fusion (concat)	87.4	7.2	0.21	89.2	Limited temporal cross-modal modeling
Late Fusion (gating)	88.2	6.8	0.20	91.0	Less adaptive under partial occlusion

## Data Availability

The data presented in this study are available on request from the corresponding author.

## Abbreviations

The following abbreviations are used in this manuscript:

UAV	Unmanned Aerial Vehicle
MFCN	Multimodal Fusion Cooperation Network
DRL	Deep Reinforcement Learning
MPC	Model Predictive Control
JT	Manual Teleoperation
AO	Autonomous Optimization
UM	Unimodal Shared Autonomy
HM	Heuristic Multimodal
HIL	Hardware-in-the-Loop
ROS	Robot Operating System
IMU	Inertial Measurement Unit
CNN	Convolutional Neural Network
LSTM	Long Short-Term Memory
PPO	Proximal Policy Optimization
NASA-TLX	NASA Task Load Index
